# Bioprinted Membranes for Corneal Tissue Engineering: A Review

**DOI:** 10.3390/pharmaceutics14122797

**Published:** 2022-12-14

**Authors:** Amin Orash Mahmoud Salehi, Saeed Heidari-Keshel, Seyed Ali Poursamar, Ali Zarrabi, Farshid Sefat, Narsimha Mamidi, Mahmoud Jabbarvand Behrouz, Mohammad Rafienia

**Affiliations:** 1 Department of Chemistry and Nanotechnology, School of Engineering and Science, Tecnologico de Monterrey, Monterrey 64849, NL, Mexico; 2 Department of Tissue Engineering and Applied Cell Sciences, School of Advanced Technologies in Medicine, Shahid Beheshti University of Medical Sciences, Tehran 1434875451, Iran; 3 Biosensor Research Center, Isfahan University of Medical Sciences, Isfahan 8174673441, Iran; 4 Department of Biomedical Engineering, Faculty of Engineering and Natural Sciences, Istinye University, Istanbul 34396, Turkey; 5 Department of Biomedical and Electronics Engineering, School of Engineering, University of Bradford, Bradford BD7 1DP, UK; 6 Interdisciplinary Research Centre in Polymer Science & Technology (Polymer IRC), University of Bradford, Bradford BD7 1DP, UK; 7 Translational Ophthalmology Research Center, Farabi Eye Hospital, Tehran University of Medical Sciences, Tehran 1985717443, Iran

**Keywords:** corneal tissue engineering, epithelium, stroma, endothelium, 3D bioprinting

## Abstract

Corneal transplantation is considered a convenient strategy for various types of corneal disease needs. Even though it has been applied as a suitable solution for most corneal disorders, patients still face several issues due to a lack of healthy donor corneas, and rejection is another unknown risk of corneal transplant tissue. Corneal tissue engineering (CTE) has gained significant consideration as an efficient approach to developing tissue-engineered scaffolds for corneal healing and regeneration. Several approaches are tested to develop a substrate with equal transmittance and mechanical properties to improve the regeneration of cornea tissue. In this regard, bioprinted scaffolds have recently received sufficient attention in simulating corneal structure, owing to their spectacular spatial control which produces a three-cell-loaded-dimensional corneal structure. In this review, the anatomy and function of different layers of corneal tissue are highlighted, and then the potential of the 3D bioprinting technique for promoting corneal regeneration is also discussed.

## 1. Introduction

The cornea, which is located in the anterior part of the eye, is a transparent layer and acts as the window of the eye [1,2,3]. The corneal structure contains three transparent layers, and two membranes [2]. The corneal structure transfers light into the eye’s environment and protects the eye’s structure from mechanical or chemical environmental injuries, UV light, and infection [4]. Corneal dysfunction causes corneal visual loss [5].

Corneal surgery and corneal transplantation are well-known therapies for corneal blindness [5]. According to the World Health Organization (WHO), about 10 million patients globally need healthy corneal donation [1]. Additionally, over 40,000 corneal transplantations are carried out in the United States annually [6]. However, corneal transplantation displayed several drawbacks including shortness of high-quality donor corneas, expensive surgery, and rejected tissue due to the immune system and weakness for long-term transplantation [7]. In addition, because aging diminishes the function of endothelial cells, the quality of the transplanted cornea is of utmost importance [8]. Furthermore, the tissue becomes ineligible for corneal transplantation by therapies that alter the corneal structure to improve vision, such as LASIK [9]. Scientists are utilizing stem cells and tissue-engineering techniques to generate bioengineered cornea, or even individual corneal layers, to address the shortage of eligible corneas for donation [10,11,12].

Tissue engineering utilizes cells, bioactive macromolecules, and scaffolds, or a blend of the mentioned factors [13,14,15,16,17]. Human corneal cell keratoplasty (HCCK) was recently chosen as an advanced corneal surgery technique. The HCCK technique includes transparent carriers to improve human corneal cell behavior [18,19,20]. These lamellar keratoplasties and tissue-engineered full-thickness are recognized as successful transplantations [5]. Although donor corneas are used in these approaches, they still possess some challenges such as allograft tissue availability and rejection [21]. Results have shown that the proliferative ability of cultured human corneal cells can be preserved; thus, cornea tissue engineering (CTE) is recognized as a suitable approach for reconstructing corneal damage [22]. A recent study revealed that human corneal cells (HCCs) have adequate efficacy for cell propagation, but they might show low biocompatibility, weak light transmittance, and poor mechanical properties [23,24,25]. There are several methods of producing tissue-engineered scaffolds that completely resemble corneal structures [9,26,27,28,29]. Among them, 3D bioprinting technology is one of the potential approaches for producing artificial target tissue scaffolds. For example, the advantage of choosing this method in scaffold construction is the induction of the natural process during embryogenetic tissue formation and imitation [30,31,32]. Overall, 3D printing is attractive due to its high spatial resolution, and the simultaneous processing of cells and materials [33]. The conventional 3D printer consists of a classic inkjet, nozzles, and printer heads with material loaded into the cartridges as bioinks [34,35,36,37,38,39]. Thus, this review paper will highlight the corneal anatomy and different corneal layers’ functions, ocular disorders, and a summary of different approaches in scaffold constructions with a specific emphasis on 3D printed corneal tissue-engineered scaffolds.

## 2. Corneal Anatomy and Physiology

**Figure 1:**
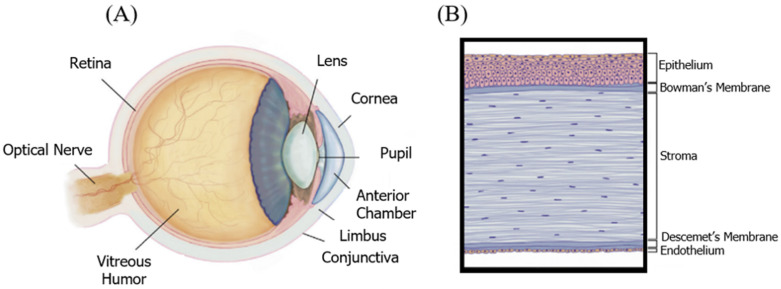
(**A**) The anatomy of the eye, cornea. (**B**) The cornea is an optically transparent multilayered structure consisting of three cell layers and two membranes. Adopted and modified from [1] (Chapter 67) with permission.

The cornea, known as the window of the eye, is optically transparent, including a special structure that is avascular anatomically. This dome-shaped and specialized tissue is located in the anterior part of the eye. Two major roles of the cornea are protecting the eye from harsh environments, and transmitting over 80% of light to inner portions (Figure 1A) [23]. As is evident in Figure 1B, the cornea is composed of three arranged and transparent layers, and two membranes: The cornea includes the outermost layer of epithelium, stroma, and the innermost layer of endothelium. Additionally, the epithelium and stroma are separated by Bowman’s membrane. However, the stroma and endothelium are separated by Descemet’s membrane (Figure 1B) [22]. Furthermore, the cornea acts as the last superficial barrier of the eye, providing safety from external potential dangers, and infections [40]. Moreover, to maintain and protect the integrity of the eye surface, corneal nerves play a vital role [23]. Consequently, corneal regeneration is obtained by nerve density, and corneal sensation factors after transplantation [4].

In addition, the aqueous humor is located at the eye’s surface, and the function of the cornea depends on its malleability [41]. Moreover, it should be noted that the tear film is placed in the outermost portion of the eye, and acts as a reservoir for antibacterial and growth factors [9]. Additionally, one of the most critical roles of tear film in maintaining homeostasis, proliferation, and repair is covering the corneal surface. The anatomical importance of the cornea, which includes five transparent and arranged layers, corresponds to a wide-angle lens [13].

### 2.1. Corneal Epithelium

The epithelium is the outermost layer of the corneal tissue, and acts critically in the refraction of light into the eye [42,43,44]. The epithelium is a highly innervated tissue with nerve endings terminating at corneal epithelial layers [45]. The epithelium is a multilayered tissue and has five cell layers which occupy 10% of the corneal structure, and is about 50 μm thick [22]. The epithelium, a biological barrier, is responsible for the transfer of all soluble constituents and water out or into the stroma to maintain proper corneal light transparency, providing a smooth layer [23]. There are three cell types in the epithelial layer of the cornea. These cell types consist of 3–4 layers of flattened squamous cells, 1–3 layers of wing cells, and a single layer of columnar basal cells. It should be noted that these cells are held together by tight junctions [1]. These cell types are regenerated every 7–10 days continuously by the limbus stem cells (LSCs) [46].

There are some challenges in the regeneration of the epithelial layer by tissue engineering approaches, such as mimicking its arranged complexity, maintaining integrity as a sufficient barrier, and replacing epithelial cells continually [47,48,49]. In general, the epithelial layer, as the outermost layer, can keep the eye safe from mechanical damage, infection, and injuries [4]. In addition, it has a role in protecting the retina from UV damage [4].

### 2.2. Corneal Stroma

The stroma occupies 90% of the corneal tissue and 5% of corneal keratocyte cells (CKCs), and is an acellular layer but also a dense connective layer derived from neural crest cells [40]. The stroma comprises over 200 noncellular collagenous lamellae that are fully uniform, small, and aligned collagen fibers [7]. When injuries occur, flattened fibroblasts are activated. These lie quiescently, typically to produce collagen, then stabilize collagenous lamellae, and secrete the stromal components [2]. There are two important properties of a healthy stroma layer: optical transparency, and suitable mechanical strength [12,13,14]. Optical transparency is needed for biophysical properties, and suitable mechanical strength can be decreased when this organized structure is disturbed. Light transmittance can be reduced as a result of stromal damage and disruption. The stroma expresses two major challenges for the tissue engineer: equal mechanical stability, and high optical transparency [50].

### 2.3. Corneal Endothelium

Although the endothelium is the thinnest layer of the corneal tissue, it is important for maintaining function, and the ability to maintain corneal reproduction [1]. It is necessary to maintain dehydration by keeping optimal optical clarity [51]. Originally, the human endothelial cells (HECs) consist of about 5000 cells/mm^2^, while the number of HECs shows loss with increasing age. In general, the major challenge for tissue-engineered transplantation is the HECs cell number of out 2500 cells/mm^2^ [52]. The endothelium functions as a leaking pump of the corneal structure by leaking from the stroma layer in the presence of excessive stromal hydration (above 80%) [53]. The pumping-leak function process contains Na^+^ and K^+^-ATPase pumps that occupy the basolateral membrane. The main function of pumping-leak is to maintain stromal relative dehydration through transporting ions and water from the stroma to the tear film and aqueous humor [54,55,56]. The main characterization challenge is the efficiency measurement of the transplanted HECs [57]. There are some selective glucose transporters in this layer, permitting nutrition transformation from the aqueous humor to feed the epithelial and CKCs. Therefore, the main function of the endothelial layer is optical transparency with regulated hydrophilic proteoglycan and collagen interfibrillar spacing. In addition, endothelial distortion might lead to a loss in pump function [58].

## 3. Cells

### 3.1. Epithelium Cells

**Figure 2:**
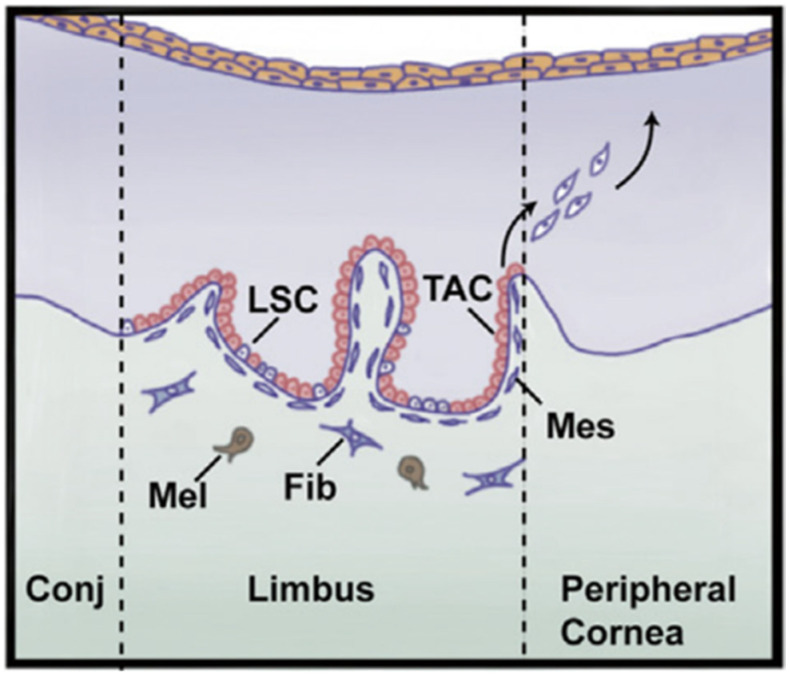
The major role of the limbus is to regenerate epithelium where the limbal stem cells (LSCs) reside. LSCs produce transient amplifying cells (TACs) that have a significant proliferation potential. Then, TACs migrate to epithelium which is responsible for producing epithelial cells and is replaced. Mes = mesenchymal cell, Mel = melanocyte, Fib = fibroblast, Conj = conjunctiva. Adopted and modified from [1] (Chapter 67) with permission.

The corneal epithelial cells function as a physical barrier that resists the outer environment to maintain a healthy stroma layer. This effective corneal cell layer has a continuous turnover, with a lifespan of approximately 7 to 10 days (Figure 2). This turnover function is well described by the XYZ hypothesis [1]: X, the basal epithelial cells form the layer capable of proliferation properties; Y, migration centripetally of peripheral cells of new basal cells from the limbus to the cornea; and Z, loss of the epithelial cells from the surface. Generally, the epithelial cells shed constantly, and are substituted by a new cell sheet [59]. X + Y = Z describes the corneal epithelium’s maintenance function: cell loss and replacement. These three stages describe the complete corneal wound healing process: Z represents the epithelial cell loss from the limbus, step Y describes the covering of the surface by the wound surface, and lastly, in the final step X, proliferation provides cells with the ability to replace the epithelial tissue. As a result, the intensity of the centripetal movement and enhancement of proliferation ability are reasons to promote corneal wound healing [60].

### 3.2. Stroma Cells

The corneal stroma consists of both extracellular and cellular components [61]. Cellular components of the mature corneal stroma are CKCs. CKCs have a dendritic morphology and are responsible for the maintenance of the ECM of the stroma. CKCs generate keratocan and lumican, and they are the key factors in maintaining the shape and transparency of the stroma. These small leucine-rich protein family members (keratocan and lumican) are the most important keratan sulfate proteoglycans in the corneal stroma [62,63,64]. Keratocan is solely found as a proteoglycan in the cornea, while lumican may also exist in various tissues as a glycosylated protein [33]. Both keratocan and lumican interact with collagen fibrils, and regulate the structure of this tissue to fit within their limits for specific properties [4]. Based on previous evidence, keratocan plays a crucial role in preserving the corneal structure [9].

Wound healing processes change the dendritic morphology of CKCs to be fibroblastic in appearance [65]. Two important functions of the keratocyte—the expression of keratocan and keratan sulfate synthesis—are decreased during the fibroblast/myofibroblast transformation [9]. Both isolated keratocytes from the corneal stroma and cultured keratocytes exhibit fibroblastic/myofibroblast phenotypes, and, meanwhile, show decreased keratocan expression and keratan sulfate synthesis, similar to in vivo wound healing [66]. This demonstrates that keratocan can be regarded as an indication of the native keratocyte phenotype [2].

### 3.3. Endothelium Cells

**Figure 3:**
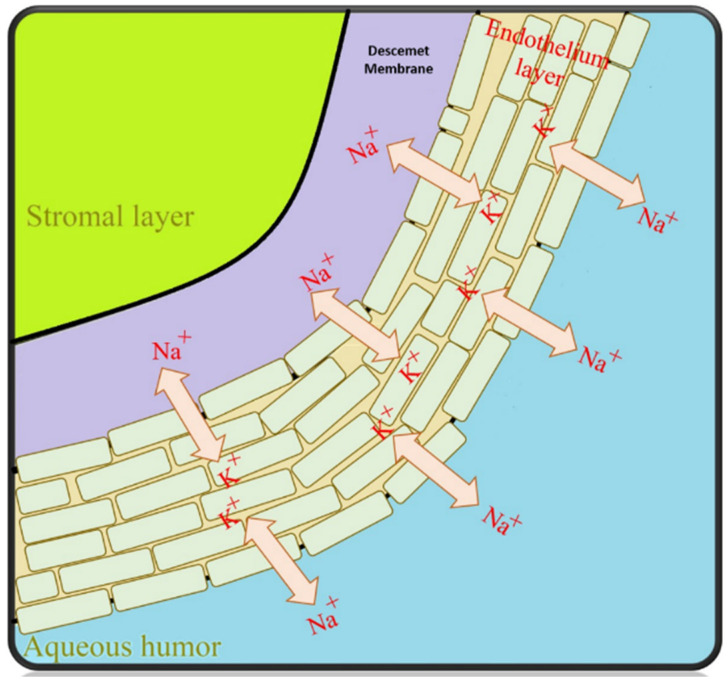
The corneal endothelial “pump-leak” hypothesis illustrated—basic principles [68].

The key function of the endothelial cells is to pump excess fluid from the stroma and epithelium into the superficial layer of the cornea to maintain optimum corneal nutrition, and hydration [67]. This is recognized as the “pump-leak hypothesis”, preserving the cornea in a dehydrated state. It is worth mentioning that the hydration stage plays an important role in optical transparency (Figure 3) [60]. Endothelial cells are responsible for transporting proteins from inner layers using Na/K ATPase pumps. Thus, this gradient provides an osmotic pressure to maintain corneal stroma hydration, which is essential for endothelial cell growth [68].

## 4. Corneal Scarring

Understanding the processes of deficiency or disease in almost any aspect of the visual system necessitates an intensive investigation of the structural foundations of the cornea, hence necessitating a considerable emphasis on individualized medical and surgical regeneration therapy. Vision impairment and obstruction of light to the eye have revealed a lot of biological information about ophthalmic diseases, ranging from damaged superficial layers and limbal cells to corneal injuries [4].

### 4.1. Keratoconus

Overall, keratoconus involves a general weakness of the connective tissue of the cornea. It is a progressive, noninflammatory corneal dystrophy resulting in thinning and protrusion of the cornea, changing it from a dome shape to a conical shape with gradual bulging [69,70,71]. Initially, patients experience blurred vision with the same symptoms as irregular astigmatism and refractive defect [72,73,74]. Vision is obscured as keratoconus progresses. The extent of vision impairment is subject to the degree of progression.

As keratoconus progresses it can be more easily diagnosed, as patients experience impaired night vision, photophobia, severe headaches due to eye strain, and eye itching. Usually, the condition is bilateral, and begins in the early teenage years. Corneal scarring is seen in advanced keratoconus stages, and can contribute to further vision loss until it eventually progresses to the point that corneal transplantation is critical to repair vision [75,76,77]. Keratoconus is responsible for stromal scarring, axial thinning, the disintegration of the epithelial basement membrane, and breaks in the Bowman’s membrane. According to the reported clinical case studies, the progression of keratoconus typically alters inevitable astigmatism from regular to irregular [4].

### 4.2. Dry Eye Disease

Dry eye has a wide range of eye surface diseases. According to a study reported in the 2007 international dry eye workshop, dry eye is a multidimensional disease, and its symptoms include tear instability, visual disturbance, eye discomfort, and potentially ocular surface damage [78]. According to data from previous studies, approximately 4.91 million Americans suffer from dry eye disease. Furthermore, there are tens of millions of less severe symptoms that can lead to dry eye failure if they are not followed up, which can trigger irritation—such as extended use of visual display terminals, or contact lens wear [79]. The pathophysiology involves either increased tear evaporation, decreased tear secretion, or both, resulting in hyperosmolarity of the tear film, and ocular surface inflammation. Corneal epithelial integrity can be seen in dry eye disease in its moderate to severe forms disrupted with punctate epithelial erosions; these erosions are detectable with fluorescein staining. The most common treatment for moderate to severe dry eyes is tearing supplementation, anti-inflammatory drops, eyelid hygiene, punctual plugs, and oral tetracycline [80].

### 4.3. Bacterial Keratitis

Bacterial keratitis is well-known as a devastating infection of the cornea, which can occur when the ocular defense is damaged. As a result, its spread causes inflammation, and gradual loss of vision. It is important to note that epithelial defects and decreased corneal sensitivity are prompting factors for severe ulcers, stromal necrosis, and bacterial growth. Failure in the protective mechanism and lack of ocular surface integrity causes the penetration of bacterial microbes into the cornea [81]. The most common causative organisms of bacterial keratitis include *Staphylococcus aureus*, *Streptococcus pneumonia*, and *Staphylococcus epidermidis* [82].

### 4.4. Light and Chemical Injuries

Clinically, exposure to UV light from a light source can damage the corneal epithelium, and cause fluorescein staining. Snow blindness, tanning bed use, direct lightening, and direct observation of the sun are some of the major causes of lesions on the corneal surface. Other important factors (such as chemical burns) can cause weakness in the cells of the corneal surface and affect the epithelial regeneration, even leading to potential blindness [83]. There are currently common treatments for superficial diseases that increase the ability of the corneal epithelium to recover. For extreme cases, cell-based restorative and repopulation treatments are necessary [21], which include techniques such as stem cell transplants, allografts, and limbal autografts to promote re-epithelialization [84].

### 4.5. Corneal Abrasion and Foreign Body

Corneal abrasions are more common in patients with symptoms in the epithelial layer, as they are more susceptible to injury. Abrasions typically occur with a range of symptoms, such as foreign body sensation, pain, tearing, sensitivity to light, and decreased vision, and it should be also noted that patients typically present with a history of trauma [85]. The presence of a foreign body within the corneal calls for immediate action to avoid permanent scarring, and serious loss of the epithelial cell surface. A deep wound with infected foreign material is likely to result in severe complications, initiating traumatic iritis, recurrent erosion syndrome, bacterial keratitis, and corneal ulcers [86].

## 5. Three-Dimensional Bioprinting

**Figure 4:**
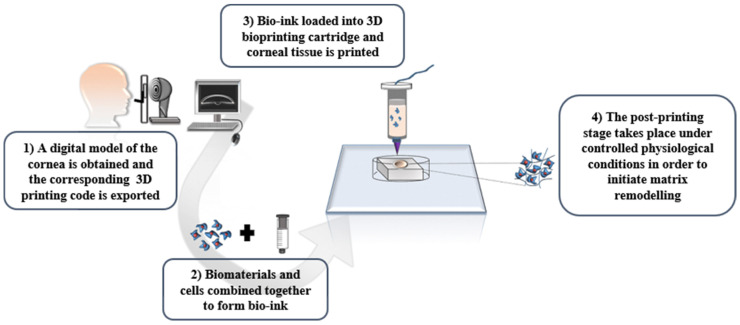
The path of making patient-specific devices via 3D printing [33] with permission.

In general, the created scaffold structure is similar for all 3D printed models (Figure 4). First, the creation of a high-quality 3D model from the desired object is required. Then, the 3D structure should be printed in 2D layers of thickness, defined by the 3D image. The data will form the structure for layer-by-layer printing by transferring the command to the printer’s desktop. The flexible manufacturing process allows it to be provided to the targeted tissue. In addition, graphical methods can be designed, including computer-aided design (CAD), and magnetic resonance imaging (MRI) of a structure similar to the data received from patients [33].

Another consideration is bioprinting, which uses cellular encapsulated biological materials as bioinks [87]. Scaffolds printed with a cell are produced in situ. In this circumstance, the printing process must be carried out in disinfected conditions, and be compatible with the cell. The importance of maintaining structure and having mechanical properties in the printed structure are factors that limit the selection of cell-compatible materials [88]. It is also important to select the appropriate rheological parameter to reduce the shear pressure, which is required for the printing parameters [89]. Nevertheless, cell-loaded bioprinting reduces the resolution of the printed substrate. Secondly, the need to increase the cell density ratio relative to the surface area is of critical importance [90]. It should be noted that a healthy threshold of cell density in solid organs is considered to be about 10^9^ to 10^10^ cells per cell culture well. Up until now, bioprinted hydrogel scaffolds only had a cell density ranging between 10^5^ and 10^7^ cells per cell culture well [91].

**Figure 5:**
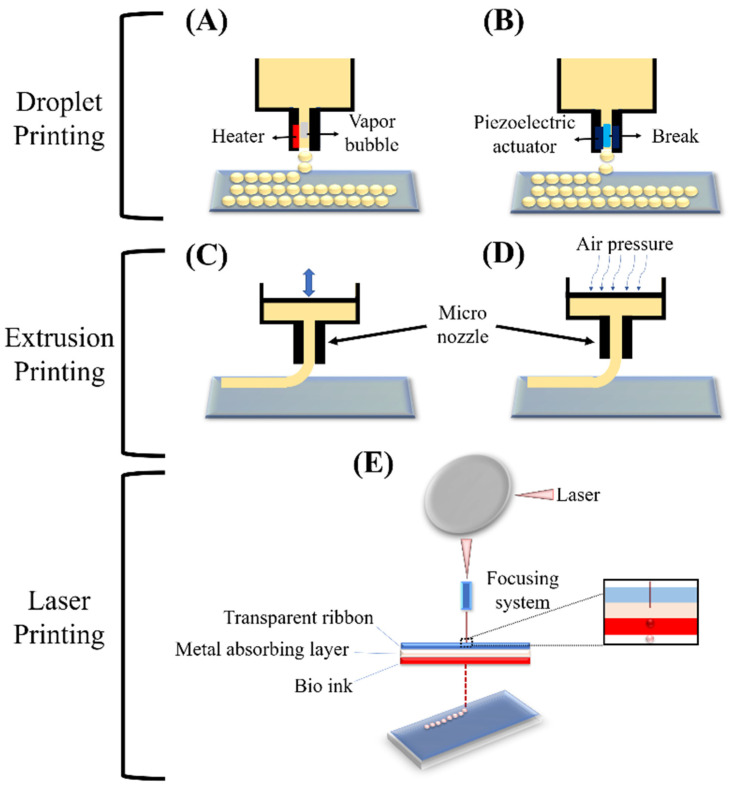
Schematic of the basic techniques of 3D printing: Droplet printing (thermal (**A**), piezoelectric (**B**)), Extrusion printing (piston (**C**), pneumatic (**D**)), and Laser printing (stereolithographic (**E**)) [100].

Bioprinting methods have successfully been applied by several researchers, and have demonstrated the reliable properties of bioprinting techniques to generate ex vivo constructs and membranes [92]. For example, scientists have obtained noticeable features by a microextrusion approach to produce a proper replacement for neural studies, generating 3D models of interacting human endothelial cells (HECs), and cancer studies by using a laser-based method, or even recreation of native ECM of cartilage by a droplet-based technique [93,94,95]. As a top-down approach, bioprinted scaffolds are known as a biofabrication technology for fabricating several types of ex vivo membranes and tissues artificially by consecutive deposition of cell-loaded layers [96,97,98,99]. Different approaches can be applied for fabricating bioprinted scaffolds such as laser-based, droplet-based, and extrusion-based techniques (Figure 5) [100]. These bioprinting techniques are compatible with several kinds of bioinks which can be crosslinked in different ways. However, optimizing bioink, according to the requirements of each of these bioprinting techniques, is associated with different challenges. An extrusion-based method is the most popular in comparison with other types of bioprinting approaches [101,102,103], since it is compatible with most injectable hydrogel platforms for biomedical engineering, and regeneration medicine applications [93,94,104]. In brief, this method contains pre-polymerized bioink which is extruded through a single nozzle under pressure. The pressurized air can be applied to the printer head to produce a 3D construct by extruding printing material layer by layer.

In addition to these three bioprinting methods, recent studies have demonstrated two more techniques that have illustrated interesting results. These techniques can be categorized into two major categories. Among these techniques, one involves using photocurable gels, and another is based on applying thermosensitive and natural (such as collagen) bioinks [105,106,107,108]. As a photocurable gel, both poly (ethylene glycol) diacrylate (PEGDA) and gelatin methacrylate (GelMA) can be crosslinked using lithium phenyl (2,4,6-trimethyl benzoyl) phosphinate (LAP) as a crosslinker under visible light. Bernal et al. [105] fabricated a GelMA-LAP bioink system to produce 3D printed osteogenic models by employing the volumetric method, setting 2D light with rotating and synchronously irradiated patterns. It should be noted that the volumetric bioprinting technique enables geometrically constructed production which aims to create centimeter-scale constructs at an unprecedented printing rate, opening new possibilities for upscaling the creation of the hydrogel-based structure. The result showed that the polymer was not cured evenly and only in some parts of the structure, preventing the gelation threshold as a result of increased absorption. Looking on the bright side, after fourteen days of continuous cell culturing, the tissue-engineered construct revealed enhanced alkaline phosphatase (ALP) expression, and mineral deposition. Another study by Grigoryan et al. [106] generated multi-vascular and intravascular structures via photopolymerizable gel with the addition of food dye for the stereolithography approach. Both studies illustrated that it is possible to fabricate a complex 3D construct via these methods, and demonstrate new possibilities for fabricating corneal tissue suitable for tissue transplant applications. The second category of bioprinting methods involves applying thermosensitive and natural bioinks [107,108]. Skylar-Scott et al. [107] generated a 3D printing scaffold with the sacrificial writing into functional tissue (SWIFT) approach. In the SWIFT technique, a high volume of cells are transferred into the engineered ECM during the bioprinting procedure. This technique contains a sacrificial gel that contains the cells. This gel is printed and after printing it is liquified by melting at room temperature; thus, the gel is removed creating a path for the medium to flow. The results have shown that after eight days of cell seeding cardiomyocyte cells showed a beating function, proving the successful functional application. Another study which was designed in the reverse order, in comparison to SWIFT, utilized freeform reversible embedding of suspended hydrogels (FRESH), supporting the 3D printed structure during the process which revealed positive results after 14 days of cell seeding [108]. Thus, the obtained results can be used for human corneal generation.

### Corneal Bioprinting

In general, corneal bioprinting offers a wide range of possibilities to address current challenges and requirements of corneal tissue regeneration (i.e., controllable structure and properties, similar mechanical strength to withstand environmental as well as structural pressure, and fabricating a fully-organized corneal construct) [100]. As was addressed in Section 2 and Section 3, the corneal structure consists of three transparent layers. The thickest layer of the cornea is the stromal layer (~500 µm), and the epithelium and endothelium are both delicate in structure (<50 µm) [109]. The stromal layer occupied over 90% of the corneal structure, and functionally is the most important tissue in CTE due to its transparency (as a result of aligned collagen lamellae and proteoglycan expressions) and mechanical performance (due to cross-linked collagen fibrils) [110]. In addition, the peripheral and central sections of the stromal layer show different mechanical properties, which play a crucial role in the orientation of collagen content, and corneal cell differentiation and alignment [111]. Therefore, to enhance the functionality of the stromal part, it is crucial to simulate the micro and macrostructure since the physical, mechanical, and chemical properties of the tissue-engineered structure directly and noticeably affect biological factors [9].

According to current studies, various bioprinting techniques for CTE have been considered (Table 1) [33,67,112,113,114,115,116,117,118,119,120,121]. Sorkio et al. [112] analyzed the feasibility of a laser-based bioprinting method to generate corneal tissue. In brief, the collagen type-I cell loaded enhanced LSCs cell attachment and proliferation. Although it is possible to print a tissue-like stromal layer, the tissue-engineered structure may not be appropriate in its structure and mechanical properties because of the very sensitive nature of CKCs. Additionally, the fabricated scaffold did not have proper transparency for corneal demands. Another study by Isaacson et al. [33] reported preparing bioink consisting of alginate-collagen type-I and CKCs. The prepared ink was injected into a 3D mold made by acrylonitrile butadiene styrene (ABS), and using the FRESH method. The final result was a structure similar to the 3D structure of corneal, with corneal tissue. Even though the produced construct showed suitable transparency, it could not support CKCs properly and the cells could not reach a dendritic shape.

The vitality and proliferation of CKCs are challenging, since this sensitive type of cell simply converts to scar-inducing stromal fibroblasts at non-desirable conditions. Recently, this challenge was overcome with the droplet-based printing technique, since it is more compatible with cells relating to laser-based or extrusion-based printing techniques. Campos et al. [113] printed collagen-type I-agarose bioink corneal construct with CKCs encapsulation to produce a dome-shaped structure in a layer-by-layer manner. The obtained results illustrated cell vitality and proliferation similar to the control sample, and showed positive expression for both lumican and keratocan markers.

From an anatomical standpoint, the stromal layer is difficult to regenerate with current techniques due to its highly complex microstructure, and it being made of randomly oriented collagen lamellae [120]. Additionally, the stromal mechanical strength and light transmittance performance are completely related to the stromal unique structure, which is fabricated from randomly oriented collagen fibrils [2]. Current research based on the fabrication of aligned PCL-PEG fibers with the incorporation of limbal stem cells (LSCs) and 15% GelMA gel to fabricate corneal tissue-engineered construct showed better mechanical strength with improved suture-ability, as well as improvement in expression of CKCs markers. Moreover, the scaffold illustrated high transparency and similar mechanical strength, in comparison with the native cornea [120]. Other studies have also been based on the advancement of bioprinting techniques for CTE by employing decellularized cornea gel or GelMA, showing improvement in CKCs differentiation, and better tensile strength. The printed scaffold improved filopodial elongations and phenotype maintenance similar to CKCs in vivo. The collected results motivated researchers to apply the bioprinting technique for the generation of CTE scaffolds due to their impact on architecture, transparency, mechanical strength, and cell/scaffold interactions [118,121].

After examining the aforementioned studies, it can be challenging to introduce the most promising technique to simulate corneal structure. For instance, enhanced mechanical features are possible with an extrusion-based approach, or improved elastic modulus can be achieved with a droplet-based technique; however, mechanical properties with a laser-based technique are not discussed yet. As reported in recent papers, the corneal stroma has about a 150–700 kPa elastic modulus [7]. Thus, the extrusion-based method would be the best candidate if mechanical properties have been chosen as the most important parameter. However, other properties such as microstructure and geometrical curvature are also prominent, which can be better satisfied using the droplet-based method. Furthermore, bioprinting considerably removes the possibility of human error, and it is superior to casting gels into molds in simulating native curvature of the corneal. Additionally, according to the microstructure, although it is not addressed by recent studies, future studies may be focused on the CKCs migration and orientation by employing smart fiber alignment [123].

## 6. Nanotechnology in CTE

Nanotechnology can be employed for the development of corneal scaffolds to enhance their physicochemical characteristics [9]. Nano scaffolds offer unique mechanical aspects that promote cell adhesion, proliferation, and differentiation, in addition to facilitating gas and nutrient exchange and waste removal [4]. For instance, dendrimers (~10 nm) are high-contrast polymers with a 3D ionic form, and many end groups [9]. The greatest benefits of dendritic systems are their high density of functional side chains, their capacity to manage network crosslinks, and their scalability across a broad range of sizes. It has been demonstrated that dendrimer-based hydrogels enhance the efficient healing of corneal fractures, without scarring or inflammation. Due to the ability to modulate the crosslinking process and alter the chemistry of crosslinking, it is feasible to influence the duration of resorption, and hence control the wound healing process over a longer period [124]. Thus, dendrimers are labile “smart” nanomaterials that can be employed for wound healing during long recovery periods, with a minimal likelihood of triggering an inflammatory reaction [124,125]. Combining nanotechnology and corneal tissue engineering with natural biomaterials could be a potential approach for reaching the current goal in this category [126]. For instance, to create biomaterials with the appropriate attributes, metal nanoparticles, graphene oxide, carbon nanotubes, and nanoliposomes can be combined. Enhancing the proliferation and functionality of additional stem cells is facilitated by their in situ transformation from sol to gel. Soft nanoparticles can interact with polymer chains and contribute to the hydrogel grid’s subsequent crosslinking, hence enhancing its mechanical aspects [126,127,128]. In a study, Tayebi et al. [129] produced chitosan nanoparticles into chitosan/polycaprolactone membranes yielding a biodegradable, transparent scaffold for cultivating corneal endothelial cells. The chitosan nanoparticles/polycaprolactone, which have the lowest wettability, exhibited transparency comparable to human stromal tissue. The scaffold was non-cytotoxic, and enhanced the proliferation of CECs. The biophysical results revealed that CECs adhered to the scaffold, and formed a dense monolayer. Thus, the created scaffold appears to be appropriate for corneal endothelium regeneration. In another study, Chang et al. [130] developed a novel ophthalmic formulation based on moxifloxacin and dexamethasone-loaded nanostructured lipid carriers mixed with collagen/gelatin/alginate for the treatment of a corneal disorder, particularly bacterial keratitis. The nanoparticles had the following characteristics: average size: 132.1 ± 73.58 nm; zeta potential: 6.27 ± 4.95 mV; entrapment efficiency: 91.5 ± 3.5%; and drug content: 18.1 ± 1.7%. The findings indicated that the nanoparticles could release an effective working concentration in 60 min, and sustain the drug release for a minimum of 12 h. While the samples did not show any toxicities, the substrate enhanced the cell numbers of CEpCs. An animal study confirmed that it inhibits the growth of pathogen microorganisms, and promotes corneal wound healing. The results suggest that the nanoparticle formulation may be an effective anti-inflammatory agent for CTE. The application of nanoparticles, through using the bioprinting technique, permits tailored therapy for more precise and successful disease treatment [100]. Nanotechnology is predicted to be employed in the future to personalize regenerative medicine utilizing human stem cells, and to provide therapeutic tools to maintain a healthy environment for the growth and maturation of stem cells in the damaged area [131]. However, nanotechnology research in CTE is still in its infancy, and only limited in vivo investigations are reported. The behavior of corneal cells in tissue engineering constructions in corneal injury has been widely proven in vitro, but in vivo proof-of-concept investigations are lacking, leaving many concerns unanswered.

## 7. Conclusions and Future Progress

Recently, different studies on the advancement of CTE replacements are focused on analyzing various biomaterials, and fabrication methods. Although there are several studies on this subject, the prior studies displayed a lack of understanding of the corneal function and its structure; therefore, there is still significant room for progress in mimicking the native corneal properties, such as corneal physicochemical properties. Even though different studies have shown that biomaterials might have similar mechanical, optical, and physical properties to the natural cornea, it is challenging to arrange these biomaterials into the same well-organized structures as the natural cornea. Bioprinted tissue engineering scaffolds with proper orthogonal lamellae architecture can be a crucial step for the successful fabrication of CTE scaffolds. In this regard, the fabrication of a successful CTE scaffold will be mostly dependent on generating necessary features, such as releasing important functional biomolecules to improve corneal components, cells, and nerve regeneration. Furthermore, to produce a tissue-engineered scaffold adjusting mechanical and optical features is crucial as well. Research shows that bioprinted scaffolds equipped with nanotechnology components and nanoscale characteristics can improve the potential of CTE. The ultimate aim of CTE is to improve, preserve, and restore vision by developing nanotechnology-enabled regenerative therapies to heal damaged corneal tissues based on unique patient needs.

## Figures and Tables

**Table 1:** Summary of different experimental studies based on 3D printing techniques.

Corneal Layer	Bioprinting Method	Material	Cell Source	Results	Ref.
Stroma	Extrusion	ALG, COL bioink, FRESH support	CKCs	➢ Similar structure to native cornea architecture encapsulated stromal cells under COL-based bioink ➢ Stromal cells showed high cell viability	[33]
	Laser	Matrigel, COL bioink	LECs	➢ Printed membranes showed maintaining good cell viability and positive labeling for COL ➢ Suffered from lack of sufficient transparency	[112]
	Extrusion	COL, dC	CKCs	➢ The differentiation potential of hTMSCs just observed with the Dc-COL membrane ➢ Proper mechanical flexibility ➢ Improved transparency properties of COL-Dc in comparison with COL scaffold	[121]
	Droplet	COL, AG	CKCs	➢ Keeping native keratocyte phenotype as well as proper elongation ➢ Similar transparency in comparison with the stromal layer	[113]
	Extrusion	GelMa, reinforced with PEG, PCL Fibers	LSSCs	➢ Providing an ideal environment for the preservation of keratocyte phenotype	[120]
	Extrusion	GelMa	CKCs	➢ Keratocytes showed keeping of the phenotype ➢ Similar transparency with the native cornea ➢ Adequate mechanical stability	[118]
	Extrusion	dC	CKCs	➢ The optimal nozzle diameter for bioprinting cornea-like aligned collagen fibrils ➢ The optimal nozzle diameter to preserve the morphology and phenotype of keratocytes ➢ Excellent transparency ➢ Keeping the keratocyte phenotype	[122]
Endothelium	Extrusion	Gelatin, RGD bioink, amniotic membrane dC support	CECs	➢ Enhanced cell vitality and proliferation	[119]
Epithelium	Extrusion	GelMa bioink, GelMa dome-shaped mold	CEpCs	➢ Extremely transparent curved membrane through geometric fabricated features	[117]
	Extrusion	ALG, GelMa, COL	CEpCs	➢ Good printability and high transparency ➢ Enhanced cell viability and proliferation ➢ Controllable in vitro degradability ➢ Improved epithelial cells markers	[114]

## Data Availability

The data presented in this study are available on request from the corresponding author.

## Abbreviations

Corneal keratocyte cells (CKCs), collagen (COL), limbal epithelial cells (LECs), alginate (ALG), agarose (AG), methacrylated gelatin (GelMa), limbal stromal stem cells (LSSCs), decellularized cornea (dC), corneal endothelial cells (CECs), corneal epithelial cells (CEpCs), human turbinate-derived mesenchymal stem cells (hTMSC).
